# RNA binding by ADAR3 inhibits adenosine-to-inosine editing and promotes expression of immune response protein MAVS

**DOI:** 10.1016/j.jbc.2022.102267

**Published:** 2022-07-15

**Authors:** Reshma Raghava Kurup, Eimile K. Oakes, Aidan C. Manning, Priyanka Mukherjee, Pranathi Vadlamani, Heather A. Hundley

**Affiliations:** 1 Department of Biology, Indiana University, Bloomington Indiana, USA; 2 Medical Sciences Program, Indiana University School of Medicine-Bloomington, Bloomington Indiana, USA

**Keywords:** ADAR, double-stranded RNA, MAVS, glioblastoma, RNA modification, A-to-I RNA editing, RNA-binding protein, posttranscriptional regulation, ADARB2, deaminase, AD, Alzheimer's disease, ADAR, adenosine deaminase that act on RNA, AEI, Alu editing index, COSMIC, Catalogue of Somatic Mutations in Cancer, dsRBP, dsRNA-binding protein, FBS, fetal bovine serum, IP, immunoprecipitation, RBP, LOAD, late-onset Alzheimer's disease, MAVS, mitochondrial antiviral signaling protein, qPCR, quantitative polymerase chain reaction, RNA binding protein, RIP, RNA-immunoprecipitation

## Abstract

Members of the ADAR family of double-stranded RNA–binding proteins regulate one of the most abundant RNA modifications in humans, the deamination of adenosine to inosine. Several transcriptome-wide studies have been carried out to identify RNA targets of the active deaminases ADAR1 and ADAR2. However, our understanding of ADAR3, the brain-specific deaminase-deficient ADAR family member, is limited to a few transcripts. In this study, we identified over 3300 transcripts bound by ADAR3 and observed that binding of ADAR3 correlated with reduced editing of over 400 sites in the glioblastoma transcriptome. We further investigated the impact of ADAR3 on gene regulation of the transcript that encodes *MAVS*, an essential protein in the innate immune response pathway. We observed reduced editing in the *MAVS* 3′ UTR in cells expressing increased ADAR3 or reduced ADAR1 suggesting ADAR3 acts as a negative regulator of ADAR1-mediated editing. While neither ADAR1 knockdown or ADAR3 overexpression affected *MAVS* mRNA expression, we demonstrate increased ADAR3 expression resulted in upregulation of MAVS protein expression. In addition, we created a novel genetic mutant of ADAR3 that exhibited enhanced RNA binding and MAVS upregulation compared with wildtype ADAR3. Interestingly, this ADAR3 mutant no longer repressed RNA editing, suggesting ADAR3 has a unique regulatory role beyond altering editing levels. Altogether, this study provides the first global view of ADAR3-bound RNAs in glioblastoma cells and identifies both a role for ADAR3 in repressing ADAR1-mediated editing and an RNA-binding dependent function of ADAR3 in regulating MAVS expression.

Misregulation of RNA-binding proteins (RBPs) contributes to pathogenic gene expression programs by influencing gene regulatory processes (1). ADARs (adenosine deaminases that act on RNA) are a family of double-stranded RNA (dsRNA)-binding proteins (dsRBPs) that can influence gene expression *via* binding to RNAs and/or by catalyzing the deamination of adenosine (A) to inosine (I) (2). As the conversion of A to I changes the RNA content relative to the genomic sequence, this process is referred to as RNA editing (3). Furthermore, as inosine is recognized as guanosine, A-to-I RNA editing events can impact the coding potential, splicing, stability, and translation of mRNAs as well as processing and targeting of small RNAs that arise from dsRNA precursors (2). Beyond these individual gene regulatory events, an emerging critical function of A-to-I RNA editing in mammalian cells is the ability to prevent aberrant recognition of cellular RNA by dsRNA sensors of the innate immune signaling pathway (4, 5). Consistent with these important biological functions, alterations in ADAR expression and/or RNA editing occur in several human pathologies, including many cancers, autoimmune disorders, and neuropathological diseases (6, 7).

In mammals, there are three ADAR proteins, ADAR1 (*ADAR*), ADAR2 (*ADARB1*), and ADAR3 (*ADARB2*) (8). ADAR1 and ADAR2 are ubiquitously expressed and catalyze A-to-I RNA editing at millions of sites in the human transcriptome (9, 10). ADAR3 is unique among the mammalian ADARs in that the expression of ADAR3 is restricted to the brain (11), and in addition to dsRNA binding activity, ADAR3 has been shown to bind single-stranded RNA (ssRNA) *in vitro* (12). In addition, in contrast to ADAR1 and ADAR2, ADAR3 cannot catalyze A-to-I editing *in vitro* (11, 12, 13). Furthermore, no editing events were identified in recent transcriptome-wide studies of RNA isolated from mice lacking both ADAR1 and ADAR2 (14, 15), providing *in vivo* evidence that ADAR3 is not an active deaminase.

Owing to the lack of functional deaminase activity, the biological function of ADAR3 was relatively unexplored for over 2 decades after the initial discovery of the protein. However, multiple recent studies have suggested that ADAR3 is important for both normal learning and memory (16), while aberrant ADAR3 expression occurs in several neuropathological diseases including amyotrophic lateral sclerosis (17), schizophrenia (18), autism spectrum disorder (19) and Alzheimer’s disease (AD) (20, 21, 22). While the molecular targets of ADAR3 that could contribute to these neurological defects are largely unknown, multiple studies have suggested that ADAR3 can act as a negative regulator of RNA editing (23). Studies of RNA-sequencing (RNA-Seq) data from the Genotype-Tissue Expression (GTEx) project indicate that ADAR3 transcript expression negatively correlates with RNA editing levels in the brain (24). In addition, editing of several genes, including *GRIA2, GRIA4, GRIK1, and GRIK2*, is significantly reduced in the hippocampal tissues of patients with late-onset AD (LOAD) who also have upregulated ADAR3 transcript levels, suggesting ADAR3 could function as a negative regulator of RNA editing in LOAD (21). However, the molecular mechanisms that ADAR3 employs to negatively regulate editing in LOAD and the normal brain are currently unknown.

Our previous work demonstrated that, in U87 glioblastoma cells, ADAR3 binds the pre-mRNA of an essential neuronal transcript, *GRIA2*, and blocks ADAR2-mediated editing of one specific adenosine (commonly referred to as the Q/R site) (25). In addition, the ability to bind dsRNA was required for ADAR3 to repress *GRIA2* editing. Despite this insight into the mechanism of how ADAR3 could serve as a negative regulator of RNA editing, the global impact of ADAR3 on editing of the glioblastoma transcriptome is unknown. The importance of addressing this gap in knowledge is underscored by the fact that altered ADAR3 expression has been reported in patients suffering from glioblastoma (25, 26, 27, 28).

In the present study, a global approach to identify ADAR3-bound RNAs and to examine the impacts of ADAR3 on RNA editing in U87 glioblastoma cells was performed. Over 3300 ADAR3-bound transcripts were identified, and ADAR3 expression led to altered editing of nearly half the editing sites identified in U87 cells. Consistent with previous observations for *GRIA2*, when we analyzed over 400 editing sites that exhibited reproducible changes in editing upon ADAR3 expression, most sites with reduced editing were within ADAR3-bound transcripts. Owing to the known biological intersection of innate immunity, ADARs, and dsRNA, the identification of *MAVS* as an ADAR3-bound transcript that exhibits reduced editing in ADAR3-expressing glioblastoma cells drew our attention. The mitochondrial antiviral signaling protein (MAVS) is a crucial adaptor molecule in the innate immune response pathway activated by the dsRNA sensors RIG-I and MDA5 (29). A prevailing model is that ADAR1 edits double-stranded regions of cellular transcripts to prevent aberrant engagement with dsRNA sensors (30, 31). Support for this model comes from the fact that *ADAR1* knockout mice die on embryonic day 12.5 due to aberrant immune activation, which is partially rescued by *MAVS* deletion (32). In addition, the embryonic lethality of ADAR1 deaminase–deficient mice can be fully rescued by loss of MAVS signaling (33). Even though the loss of ADAR1 leads to increased MAVS activity, no change in *MAVS* mRNA expression was reported in mice (30) or primary macrophages (34). However, ADAR1-mediated editing sites in the *MAVS* 3′ UTR have been identified and studies have suggested that MAVS expression may be posttranscriptionally regulated by these editing events as well as other RNA modifications and/or microRNAs (35, 36, 37, 38). Herein, our results add to the complex gene regulation of MAVS by revealing a unique role for ADAR3 binding to the MAVS transcript and upregulating MAVS protein expression in glioblastoma cells, independent of the editing levels within the *MAVS* 3′ UTR.

## Results

### ADAR3 binds to over 3000 transcripts in glioblastoma cells

**Figure 1:**
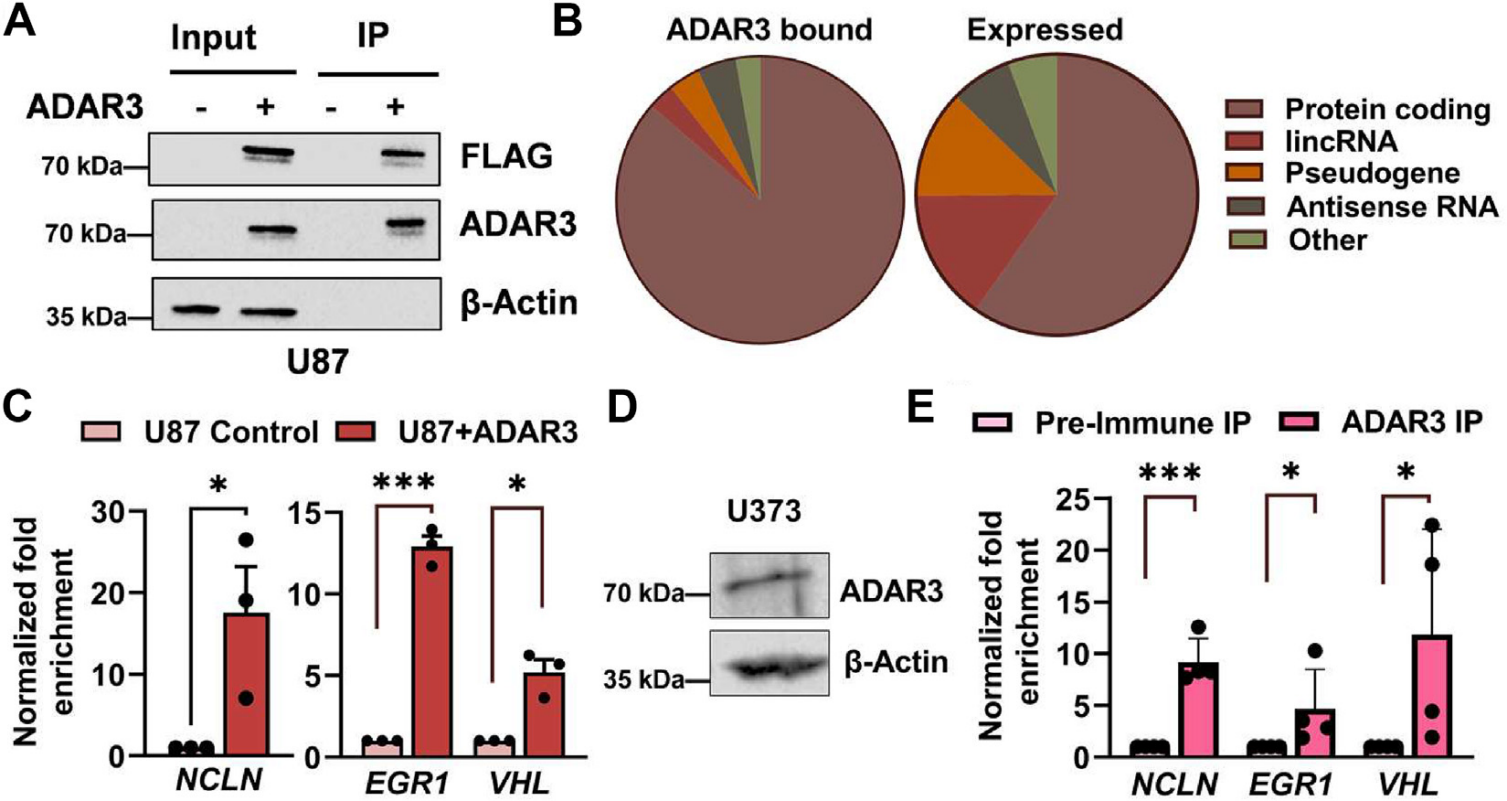
**ADAR3 binds to over 3000 transcripts in glioblastoma cells**. *A*, U87 cells transduced with retrovirus carrying neomycin resistance vector with no protein or 3× FLAG:ADAR3 expressed under the control of the CMV promoter were immunoprecipitated using FLAG magnetic beads. Input lysates from each cell line before incubation with beads (0.5% of lysate used per immunoprecipitation [IP]) and the corresponding protein immunoprecipitated (10% of each IP) were subjected to immunoblotting using antibodies against FLAG, ADAR3, and β-actin (loading control). *B*, pie chart representing the types of transcripts identified as bound by ADAR3 (*left*) and the types of transcripts expressed (reads ≥1 in at least one replicate of the U87+ADAR3 input RNA-Seq datasets) (*right*). *C*, bar graph represents the fold enrichment of cDNA present in the IPs divided by cDNA present in the input lysates from the indicated cell lines and normalized to the same ratio in U87 control cells (n = 3 independent biological replicates). The mean of the replicates is plotted with error bars representing standard error of the mean (SEM). Statistical significance was determined using a two-tailed paired *t* test, ∗*p* ≤ 0.05, ∗∗∗*p* ≤ 0.0005. *D*, lysate from glioblastoma cell line U373 was subjected to immunoblotting using ADAR3 and β-actin antibodies. *E*, bar graph represents the relative cDNA present in the ADAR3 IPs normalized to that of preimmune IPs (n = 4 independent biological replicates). The mean of replicates is plotted with error bars representing SEM. Statistical significance determined by two-tailed paired *t* test, ∗*p* ≤ 0.05, ∗∗∗*p* ≤ 0.0005.

To better understand how elevated ADAR3 expression in glioblastoma tumors impacts the transcriptome, an unbiased, global approach to identify ADAR3-bound transcripts in the U87 glioblastoma cell line was employed (Fig. 1*A*). This cell line was chosen as it has low endogenous ADAR3 expression and we previously used retroviral transduction to generate a U87 glioblastoma cell line with stable 3xFLAG:ADAR3 expression, which allowed for efficient immunoprecipitation (25). Immunoprecipitations with FLAG magnetic resin were performed for both 3xFLAG:ADAR3 cells (U87+ADAR3) and control U87 cells, which were previously generated by transducing with a retrovirus where no human gene is expressed from the CMV promoter (U87 Control) (Fig. 1*A*). As RNAs can nonspecifically associate with RNA-binding proteins after cell lysis (39), the control and ADAR3-expressing cells were subjected to UV irradiation prior to cell lysis. The covalent cross-linking also allowed for stringent washing of the immunoprecipitations to reduce the chances of capturing indirectly associated RNAs. Immunoprecipitation (IP) of ADAR3 was confirmed by immunoblotting using both a commercial FLAG antibody and a custom ADAR3 antibody (Fig. 1*A*). Total RNA was isolated from the immunoprecipitated samples and collected from each line concomitantly for sequencing the “input” RNA to control for differential gene expression between the cell lines. As ADAR3 was previously shown to bind the precursor mRNA (pre-mRNA) of *GRIA2* (25), RNA libraries were prepared using a ribosomal RNA depletion kit, allowing identification of ADAR3-bound targets that may be nonpolyadenylated. The resulting RNA-immunoprecipitation (RIP) libraries for three biological replicates of each sample were subjected to high-throughput sequencing (RIP-seq). To determine significant enrichment of transcripts in the ADAR3 IP, raw read counts were input into DESeq2 (v1.18.1) to test (IP U87+ADAR3/Input U87+ADAR3)/(IP U87 Control/Input U87 Control), using a likelihood ratio test. Transcripts with a log_2_ fold change ≥0.5 and a *p-adjusted (p-adj)* value <0.05 (Benjamin–Hochberg correction) were considered ADAR3-bound transcripts (3573 genes, Table S1*B*). A secondary bioinformatics analysis of the RIP-seq data was also performed using EdgeR (40) and identified 3596 transcripts significantly bound by ADAR3 (*p-adj* <0.05) (Table S1C). Overlap of the two methods identified 3316 ADAR3-bound targets (Table S1*A*). The ADAR3-bound RNAs are primarily protein-coding transcripts (Fig 1*B*, left pie chart), which appear to be enriched in the IPs from ADAR3-expressing U87 cells compared with the distribution of transcript types expressed in ADAR3-expressing U87 cells (Fig 1*B*, right pie chart). In addition, several other types of noncoding RNAs such as long intergenic noncoding RNAs (lincRNAs) and antisense RNAs (Table S1*A*) were also identified as ADAR3-bound transcripts.

Three additional biological replicates of the RIP assay were performed, and real-time quantitative polymerase chain reaction (qPCR) was used to validate ADAR3-bound targets identified in the RIP-seq. All three protein-coding genes (*NCLN*, *VHL*, *EGR1*) analyzed were found to be significantly enriched in IPs from ADAR3-expressing U87 cells compared with IPs from control cells (Fig. 1*C*). Using a custom ADAR3 antibody or preimmune antibody as a negative control, RIP-qPCR was also performed in a second glioblastoma cell line, U373, which expresses endogenous ADAR3 (Fig. 1*D*). All three protein-coding targets were found to be significantly enriched in the ADAR3 IPs compared with the preimmune IPs from U373 cells (Fig. 1*E*), suggesting that our RIP-seq approach identified high confidence binding targets of ADAR3 in the glioblastoma transcriptome.

### ADAR3 inhibits RNA editing across the transcriptome

**Figure 2:**
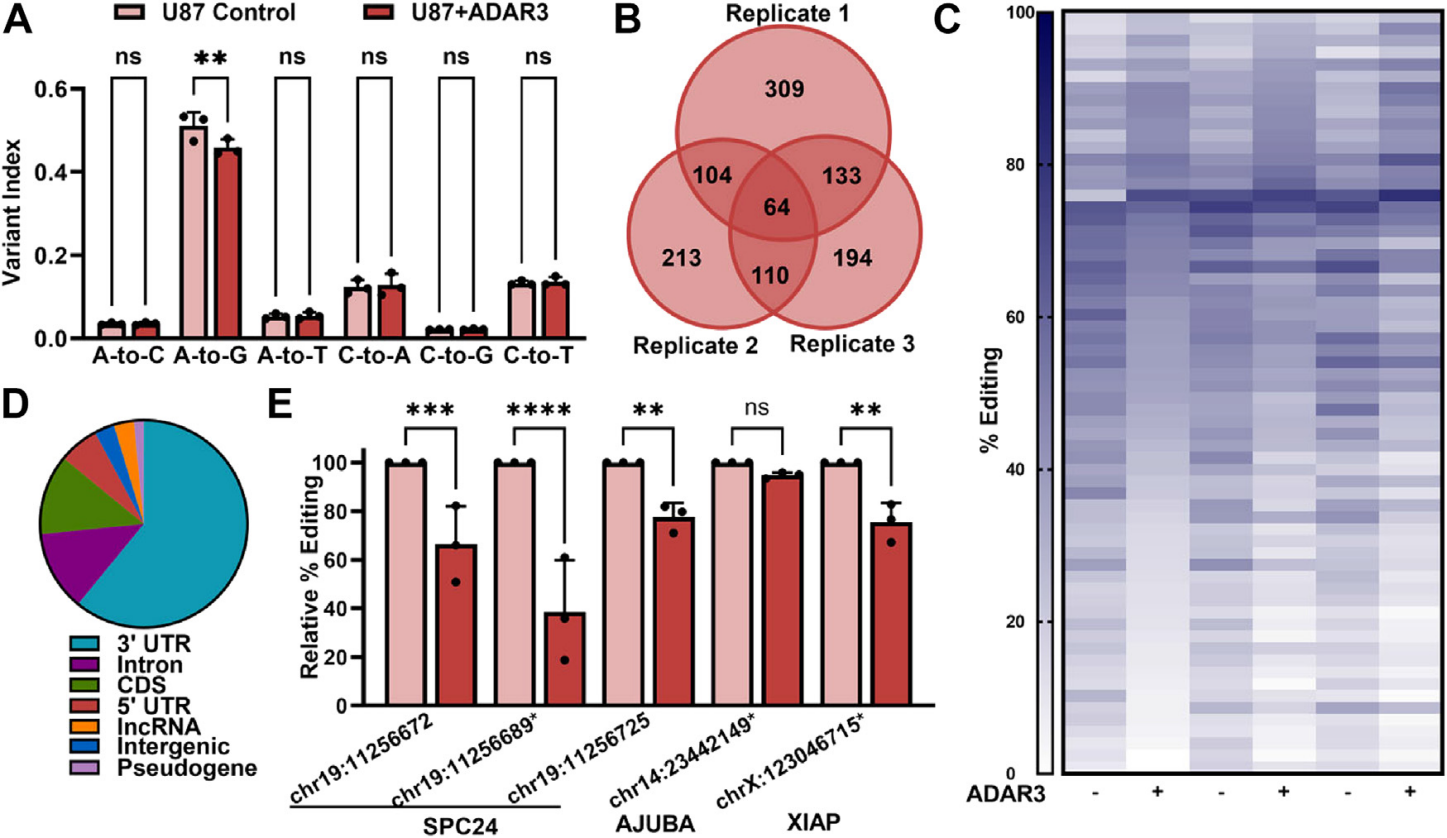
**ADAR3 represses RNA editing across the transcriptome**. *A*, variant index for ADAR3-expressing cells compared with control U87 cells. Statistical significance was determined by two-way ANOVA Sidak’s multiple comparisons test, ∗∗*p* ≤ 0.005. *B*, Venn diagram of A-to-I editing sites identified in three biological replicates of ADAR3-expressing U87 cells with ≥ |5|% change in editing upon ADAR3 expression. *C*, heatmap of the percent editing (colored in increasing shades of *navy blue*) determined from variant calling of three independent biological replicates of RNA-Seq data. Each line represents an individual editing site identified from the RNA-Seq data of the U87 cell lines indicated. *D*, pie chart representing the distribution of ADAR3-mediated differentially edited sites based on the genomic location of editing sites within transcripts. *E*, the A-to-I editing percentage was determined by a Sanger sequencing–based editing assay in ADAR3-expressing cells and normalized to that of control U87 cells. The mean of three independent biological replicates is plotted with error bars representing SEM. Statistical significance was determined using a two-way ANOVA Sidak’s multiple comparisons test, ∗*p* ≤ 0.05, ∗∗*p* ≤ 0.005, ∗∗∗*p* ≤ 0.0005, ∗∗∗∗*p* < 0.0001.

A striking characteristic of the 3316 identified ADAR3-bound target RNAs is that nearly 80% (2611) are present in REDIportal (41), a database of A-to-I editing events (Table S1*A*). We previously demonstrated that, in U87 cells, ADAR3 binding to one transcript, *GRIA2*, was important for the ability of ADAR3 to inhibit editing at the Q/R site, which is an essential editing event catalyzed by ADAR2 (42). In addition, previous global studies of editing in brain tissue revealed that ADAR3 expression negatively correlated with editing levels (24). To directly determine whether the expression of ADAR3 globally alters editing of the glioblastoma transcriptome, we determined the Alu editing index (AEI) on high-throughput sequencing data of polyadenylated (polyA+) RNA isolated from both control and ADAR3-expressing U87 cells. The AEI computational tool provides a quantification of editing levels across tens of millions of editing sites that occur in human Alu elements and was designed to identify the impacts of potential editing regulators (43). The AEI or variant index is defined as the weighted average of the A-to-G or any mismatches between a genomic reference and an RNA-Seq dataset to the total number of adenosines (or other nucleotide of interest) within the region, where the weight is equal to the coverage of each site. A significant decrease in the AEI was observed when comparing the RNA-Seq dataset of ADAR3-expressing U87 cells with control cells (Fig 2*A*). Importantly, no significant difference in the indices of other common mismatches or RNA editing sites catalyzed by cytidine deaminases was observed (Fig. 2*A*). The reduction of editing in Alu sequences, which are primarily edited by ADAR1 (44), suggests that ADAR3 can act as a negative regulator of ADAR1.

To further identify the individual transcripts and editing sites that ADAR3 alters in U87 cells, *de novo* RNA editing site identification was performed on the polyA+ RNA-Seq data using *SAILOR*, a publicly available software developed for the accurate identification of A-to-I editing sites (45, 46). The *de novo* site identification was performed on three biological replicates of RNA-Seq data from the control U87 cells. The number of uniquely mapped reads in each of the replicates was ∼80 million. Computationally identified editing sites with a confidence level of ≥0.99 and ≥20 sequencing reads in all three replicates were considered putative RNA editing sites. The *de novo* RNA editing site identification in control U87 cells yielded 1449 putative editing sites. Although single nucleotide polymorphism (SNP) information was subtracted using the *SAILOR* software, to further increase the accuracy of the RNA editing identification, second filtering of known SNPs from the dbSNP database was performed. This resulted in a refined list of 1233 candidate editing sites present in the U87 transcriptome (Table S2). Importantly, over half of these candidate editing sites (629/1233) have been reported in other studies (41) (Table S2).

To determine the consequences of ADAR3 expression on editing at these high-confidence sites, the editing levels in control and ADAR3-expressing cells were determined by identifying the number of reads with adenosine and inosine (guanosine) at each of the candidate editing sites using SAMtools (47). For accurate quantification of editing levels, only sites with ≥20 reads in all three biological replicates of RNA-Seq data from both control and ADAR3-expressing U87 cells were analyzed. Furthermore, identified changes in editing levels were limited to those that exhibited a ≥5% difference between the two cell lines. Of the 1233 editing sites identified in U87 cells, individual biological replicates of RNA-Seq data had approximately 500 to 600 sites that varied in editing levels between the ADAR3-expressing and control U87 cells (Table S3*B*). However, it should be noted that there was substantial variability in the specific sites with altered editing across the three biological replicates of RNA-Seq data (Fig. 2*B*). While nearly one-third of the differentially edited sites were common between any two individual biological replicates, using the most stringent criteria that editing sites must exhibit consistent differential editing (≥5% difference in the same direction) across all three biological replicates of RNA-Seq data revealed 64 adenosines where the editing level was significantly altered by the expression of ADAR3 (Fig 2*C* and Table S3*A*).

The magnitude of differential editing between ADAR3-expressing cells and control U87 cells varied across these 64 sites (Fig 2*C* and Table S3*A*). However, a clear trend in the differential editing was that over 70% of these sites exhibited decreased editing in ADAR3-expressing cells compared with control U87 cells (Table S3*A*). Furthermore, a similar trend of most differentially edited sites exhibiting reduced editing in the presence of ADAR3 was also observed in the ∼175 differentially edited sites that were common to only two biological replicates of RNA-Seq data (Fig. S1). The 64 adenosines with the most reproducible differential editing mapped to 57 different genes (Table S3*A*) and occur in various genomic regions (Fig. 2*D*), with the majority occurring in 3′ untranslated regions (UTRs). As ADAR1 predominantly edits 3′ UTRs (48), these data provide further evidence that ADAR3 can inhibit ADAR1 editing in U87 cells.

To validate the changes in editing observed in the RNA-Seq datasets, RNA editing was examined in independent biological replicates of RNA isolated from ADAR3-expressing and control U87 cells. Reverse transcription followed by PCR of the genes of interest and Sanger sequencing confirmed reduced editing of five adenosines in ADAR3-expressing cells compared with U87 cells (Fig. 2*E*). However, across the three biological replicates, statistically significant decreases were only observed for four of the five editing sites. It is important to note that, of these five differentially edited sites, three were observed in all three biological replicates of RNA-Seq data (chr19:11256689, chr14:23442149, chrX:123046715) while the other two significantly different editing sites in *SPC24* were only observed in two biological replicates of RNA-Seq data (Table S3*B*). Together, these data indicate that ADAR3 expression leads to altered RNA editing at hundreds of sites in the glioblastoma transcriptome.

### ADAR3 binding is correlated with reduced RNA editing

**Figure 3:**
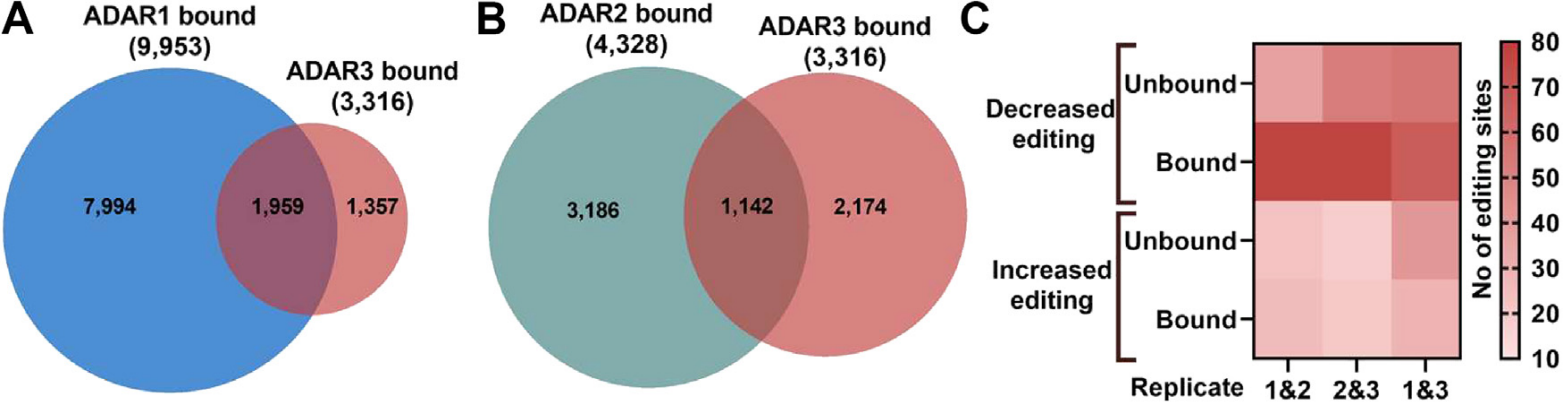
**ADAR3 Binding Is Correlated with Reduced ADAR1 mediated RNA Editing.***A*, overlap between transcripts bound by ADAR3 identified in this study with ADAR1-bound transcripts previously identified in U87 cells (44). *B*, overlap between transcripts bound by ADAR3 identified in this study with ADAR2-bound transcripts previously identified in HEK293 (50) and HeLa cells (51). *C*, heatmap represents the number of sites that exhibit ≥ |5|% change in editing in ADAR3-expressing U87 cells compared with control cells (colored in increasing shades of *red*) for the RNA-Seq datasets indicated on the *x*-axis. The heatmap is divided into an *upper panel* for those sites that exhibit reduced editing in the presence of ADAR3 and a *bottom pane*l for those sites that exhibit increased editing in the presence of ADAR3. The *upper* and *lower panels* are further binned into whether the sites occur in ADAR3-bound transcripts identified in this study.

Initial *in vitro* studies indicated that ADAR3 could bind to the same dsRNAs as ADAR1 and ADAR2, and the presence of ADAR3 resulted in reduced editing by both ADAR1 and ADAR2 (12). We previously demonstrated that, in U87 cells, ADAR3 binding to one transcript, *GRIA2*, was important for the ability of ADAR3 to inhibit editing at the Q/R site, which is edited by ADAR2 (42). As a first step toward understanding the ability of ADAR3 to bind cellular RNAs and impact editing globally, we sought to determine whether ADAR3 shared binding targets with other ADAR family members. A previously published CLIP-seq dataset from U87 cells identified 9953 transcripts bound by endogenous ADAR1 (44). Of the 3316 ADAR3-bound transcripts we identified in U87 cells, 60% (1959 transcripts) are also known ADAR1-bound transcripts (Fig 3*A* and Table S1*A*). Nearly all transcripts that can be bound by both ADAR1 and ADAR3 (92.8%,1,819/1959) are reported to be edited in REDIportal (Table S1*A*). Furthermore, 335 of the overlapping ADAR1/ADAR3-bound transcripts were previously observed to be edited in U87 cells (49) (Table S1*A*).

To date, there is no published transcriptome-wide dataset of ADAR2-bound targets in U87 cells; however, transcriptome-wide ADAR2-bound targets have been determined from both human embryonic kidney (HEK293) (50) and cervical cancer (HeLa) (51) cells that exogenously express ADAR2. Even though the cell lines have different transcriptomic profiles, ADAR3-bound RNAs in U87 cells overlap with 1142 (of 4328) (Figs. 3*B*) and 32 (of 290) ADAR2-bound targets in HEK293 and HeLa cells, respectively (Table S1*A*). Furthermore, the vast majority of the transcripts (1098/1142 and 29/32) bound by both ADAR2 and ADAR3 are edited in REDIportal (Table S1*A*). Together, these data suggest that ADAR1, ADAR2, and ADAR3 share bound RNA targets in cells, which supports the prevailing paradigm that dsRBPs largely recognize the structure of a given target dsRNA, not a specific sequence (52).

The possible collisions of all three ADARs on cellular targets impacting editing levels are consistent with the fact that dsRBPs are a major class of RNA editing regulators (53, 54). However, as dsRBPs, including ADARs, are also known to have physical interactions with other dsRBPs (2), it is possible that the impacts of ADAR3 on RNA editing we observed in U87 cells are due to physical interactions between ADAR3 and ADAR1 and ADAR2. Our previous work indicated that ADAR3 did not inhibit ADAR2-mediated editing by altering ADAR2 expression or by physically interacting with ADAR2 (55). As this current work indicates that ADAR3 can inhibit ADAR1-mediated editing, the impact of ADAR3 on ADAR1 expression was examined. Neither ADAR1 mRNA or protein expression changed in U87 cells expressing ADAR3 (Fig S2, *A* and *B*). In addition, immunoprecipitation of ADAR3 did not reveal a physical interaction with ADAR1 (Fig. S2*C*). Together, these data suggest that the impact of ADAR3 on RNA editing of the glioblastoma transcriptome is not through alterations in the levels of the active deaminases or by physically interacting with these enzymes and sequestering them from binding RNA.

To begin to understand if RNA binding by ADAR3 is important to regulate editing across the glioblastoma transcriptome, the differentially edited sites in ADAR3-expressing U87 cells were compared with the list of identified ADAR3-bound targets. As the ADAR3-bound target identification was limited to genes, the following analysis excluded intergenic sites. The sites with differential editing (≥|5|%) in each pair of replicates (Table S3, *B*–*D*) were grouped based on whether the sites have decreased (top two lines) or increased editing (bottom two lines) upon ADAR3 expression (Fig. 3*C*). These groups were further separated into two categories based on whether the site resides in an ADAR3-bound transcript (bound) or not (unbound) (Fig. 3*C*). In total, this analysis included over 400 sites and indicated that most sites with differential editing upon ADAR3 expression are sites with reduced editing that are also located in ADAR3-bound transcripts (Fig. 3*C*). The correlation of ADAR3 binding and reduced editing suggests that ADAR3 negatively regulates active ADARs by competitive binding to target RNA. While these data suggest that ADAR3 binding directly alters the ability of ADAR1 and ADAR2 to edit, there are differentially edited sites that are not bound by ADAR3. ADAR3 may also mediate changes in editing by interacting with other regulators of editing and/or altered expression of editing regulators. In this regard, we previously reported that ADAR3 interacts with ILF3 (25), which has recently been shown to bind near editing sites and negatively regulate editing (54, 56). Together, these data suggest that ADAR3 binding to transcripts or editing regulators might alter recognition of the active ADAR enzymes for the same substrates, which leads to decreased editing of the glioblastoma transcriptome.

### ADAR3 regulates expression of MAVS protein, but not mRNA

**Figure 4:**
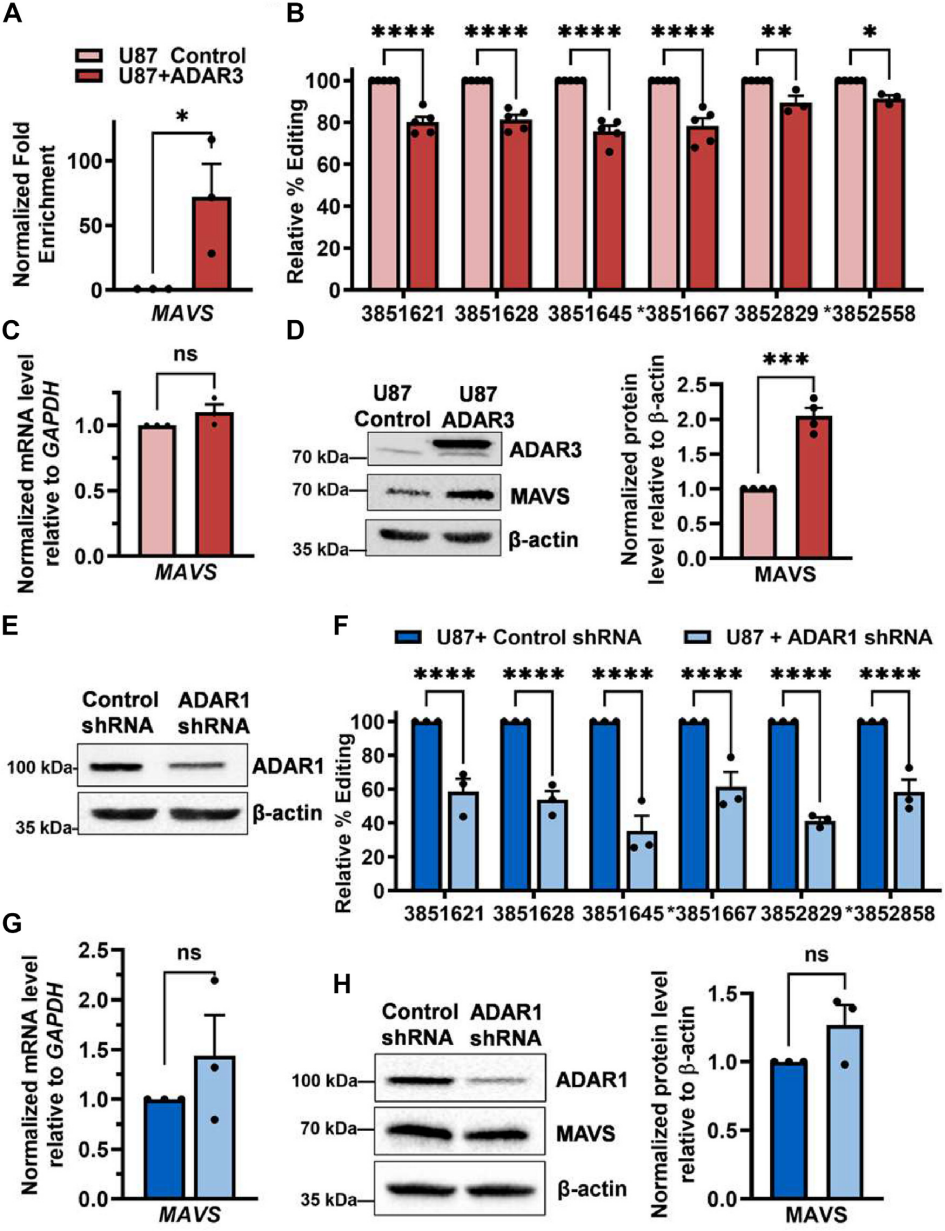
**ADAR3-mediated regulation of MAVS expression**. *A*, bar graph represents the fold enrichment of cDNA in the IPs divided by cDNA in the input lysates for the indicated cells with both ratios normalized to the same ratio in U87 control cells. The mean of three biological replicates is plotted with error bars representing SEM. Statistical significance was determined using a two-tailed unpaired *t* test, ∗*p* ≤ 0.05. *B*, the editing level at each site in the *MAVS* 3′ UTR in control and ADAR3-expressing U87 cells was determined. The numbers on *x*-axis represent the position of each editing site on chromosome 20. The data represent the percentage editing in U87+ADAR3 cells normalized to control cells in 3 to 5 independent biological replicates. Statistical significance was determined using two-way ANOVA followed by Sidak’s multiple comparisons tests, ∗∗∗∗*p* < 0.0001, ∗∗*p* < 0.005, ∗*p* < 0.05. *C*, qPCR quantification of *MAVS* mRNA level normalized to *GAPDH* in three biological replicates of control and ADAR3-expressing U87 cells. Statistical significance was determined using a two-tailed unpaired *t* test, ns = *p* > 0.05. *D*, equivalent amounts of lysate from the indicated cell lines were subjected to immunoblotting for ADAR3, MAVS, and β-actin. Bar graph represents the quantification of MAVS protein relative to β-actin for three biological replicates. Statistical significance determined by two-tailed unpaired *t* test, ∗∗∗*p* < 0.0005. E, equivalent amounts of lysates from the indicated cell lines were subjected to immunoblotting with antibodies for ADAR1 and β-actin. Blot is the representative image of three independent biological replicates. F, the editing level of the *MAVS* 3′ UTR in control and ADAR1 shRNA expressing U87 cells as mentioned in B (n = 3 biological replicates). Two-way ANOVA followed by Sidak’s multiple comparisons tests, ∗∗∗∗*p* < 0.0001. *G*, qPCR quantification of *MAVS* mRNA level normalized to *GAPDH* in U87 cells expressing a control shRNA or an ADAR1 shRNA. The mean of three biological replicates is plotted with SEM. Statistical significance was determined using a two-tailed unpaired *t* test, ns = *p* > 0.05. *H*, equivalent amounts of lysates from the indicated U87 cell lines were subjected to immunoblotting for MAVS and β-actin. Blot is the representative image of three independent biological replicates. The bar graph represents the quantification of MAVS protein relative to β-actin. Statistical significance was determined using a two-tailed unpaired *t* test, ns = *p* > 0.05.

In examining the lists of shared ADAR-bound targets as well as the transcripts that exhibit reduced editing in ADAR3-expressing cells (Table S3*A*), we noted the presence of an important transcript with known biological connections to ADARs on these intersecting lists. That transcript encodes *MAVS*, an essential protein in the innate immune response pathway (57). ADAR3 binding to *MAVS* was confirmed by performing three independent biological replicates of the FLAG-ADAR3 RIP assay followed by qPCR for *MAVS*. Compared with the IP from control U87 cells, the IPs from ADAR3-expressing U87 cells exhibited an approximately 60-fold increased enrichment of *MAVS* transcript (Fig 4*A*), confirming that *MAVS* is an ADAR3-bound target RNA. Binding of endogenous ADAR3 to the *MAVS* transcript was also observed in U373 cells (Fig. S3*A*). Together, these data indicate that *MAVS* is a target of ADAR3 in glioblastoma cell lines.

In addition to ADAR3 binding, our RNA-Seq analysis identified two sites in the *MAVS* 3′ UTR that had altered editing in ADAR3-expressing cells compared with control U87 cells (Table S3). To validate these changes, RNA was isolated from additional independent biological replicates of both control and ADAR3-expressing U87 cells and reverse transcribed, and a portion of the *MAVS* 3′ UTR was PCR amplified and subjected to Sanger sequencing. In addition to the two sites in the *MAVS* 3′ UTR that were observed in the RNA-Seq analysis of ADAR3-expressing and control U87 cells (chr20:3851667, chr20:3852558), the editing assay here identified four additional RNA editing sites in the *MAVS* 3′ UTR that all exhibit reduced editing in ADAR3-expressing cells compared with control cells (Fig. 4*B*). These data are consistent with a recent report where ADAR3 was expressed in HEK293 cells and 5425 ADAR3-bound regions were identified (50). Interestingly, in spite of the different transcriptome profile, 1372 of ADAR3 targets in HEK293 cells overlap with ADAR3-bound transcripts in U87 cells, including the *MAVS* transcript (Table S1*A*).

To determine whether ADAR3 binding and differential editing of the *MAVS* 3′ UTR alter expression, MAVS protein and mRNA levels were examined in ADAR3-expressing and control U87 cells. While no significant change in *MAVS* mRNA expression was observed (Fig. 4*C*), a 2-fold increase in MAVS protein expression was observed in ADAR3-expressing cells compared with control U87 cells (Fig. 4*D*). Together, these data indicate that ADAR3 binds the *MAVS* transcript, inhibits editing within the 3′ UTR, and ADAR3-expressing cells have elevated levels of MAVS protein, but not mRNA.

To assess if ADAR3 upregulates MAVS protein expression by inhibiting RNA editing, we directly tested whether reduced editing of the *MAVS* 3′ UTR led to increased MAVS protein levels in U87 cells. As editing in noncoding regions is performed by ADAR1 in most cells, and a previously published ADAR1 CLIP-seq study indicated that ADAR1 binds to the *MAVS* transcript in U87 cells (44), whether loss of ADAR1 leads to decreased editing of the *MAVS* 3′ UTR was examined. To generate cells with reduced ADAR1 protein expression, U87 cells were transduced with a lentivirus carrying a scrambled small hairpin RNA (shRNA) or an ADAR1 shRNA, which we previously used to reduce ADAR1 protein levels in HeLa cells. Immunoblotting of ADAR1 shRNA-expressing cells from three biological replicates revealed a decrease in ADAR1 expression compared with the control scrambled shRNA-expressing cells (Fig. 4*E*). The *MAVS* editing assay described above was performed on RNA isolated from three independent biological replicates of U87 cells expressing either the scrambled shRNA control or the ADAR1 shRNA. Quantification of the six sites in the *MAVS* 3′ UTR revealed significantly decreased editing in the ADAR1 shRNA-expressing cells compared with control U87 cells (Fig. 4*F*). Notably, reduced ADAR1 expression led to a 40 to 50% reduction in editing at each of the six sites in the *MAVS* 3′ UTR (Fig. 4*F*), while ADAR3 overexpression resulted in only a 10 to 25% reduction at each site (Fig. 4*B*).

Despite leading to a significant decrease in editing of the *MAVS* 3′ UTR, loss of ADAR1 did not result in a significant change in *MAVS* mRNA or protein expression. Specifically, both the qPCR (Fig. 4*G*) and Western blot (Fig. 4*H*) analysis indicated a slight increase in *MAVS* mRNA and protein expression upon ADAR1 knockdown; however, neither of these changes were found to be statistically significant upon analyzing three biological replicates. It is possible that MAVS expression would increase to a significant extent upon additional knockdown of ADAR1 or complete ADAR1 knockout. However, the editing levels in the *MAVS* 3' UTR are already more pronounced in the ADAR1 knockdown, yet the MAVS protein expression is more pronounced in the ADAR3 overexpression cells. These data suggest that MAVS protein expression can be regulated by ADAR3 *via* a mechanism that does not directly correlate with the extent of editing in the *MAVS* 3′ UTR.

### ADAR3 deaminase domain mutations enhance RNA binding and MAVS upregulation

Our data indicate that ADAR3 binds to the *MAVS* transcript and has a unique role in upregulating MAVS protein expression, likely independent of the ability of ADAR3 to repress RNA editing within the *MAVS 3′* UTR. To specifically test the requirements of ADAR3 to regulate MAVS protein expression, we sought to generate an ADAR3 mutant that binds RNA but not repress editing. A recent study used computational design and functional screening to engineer an ADAR3 protein that exhibited a gain of deaminase activity and contained five individual point mutations, E527Q, A389V, V485I, Q733D, and Q549R (58). Based on that study and to reduce the number of changes to wildtype ADAR3, two mutations, E527K and Q549R, were introduced into the ADAR3 lentiviral vector. The ADAR3 E527 residue is analogous to E488 in ADAR2, which, when mutated to glutamine (Q) or lysine (K), results in hyperediting activity (59). For ADAR3, E527K is one of the highly recurrent missense mutations identified in the ADAR3 deaminase domain in the Catalogue of Somatic Mutations in Cancer (COSMIC) database (60). The Q549 residue in ADAR3 is analogous to R510 in ADAR2, which has been shown to play a key role in stabilizing the orphan nucleotide during deamination in structural studies (61). In addition, this arginine residue is highly conserved in active adenosine deaminases, and mutation to glutamine in ADAR2 (R510Q) resulted in reduced editing activity (61).

**Figure 5:**
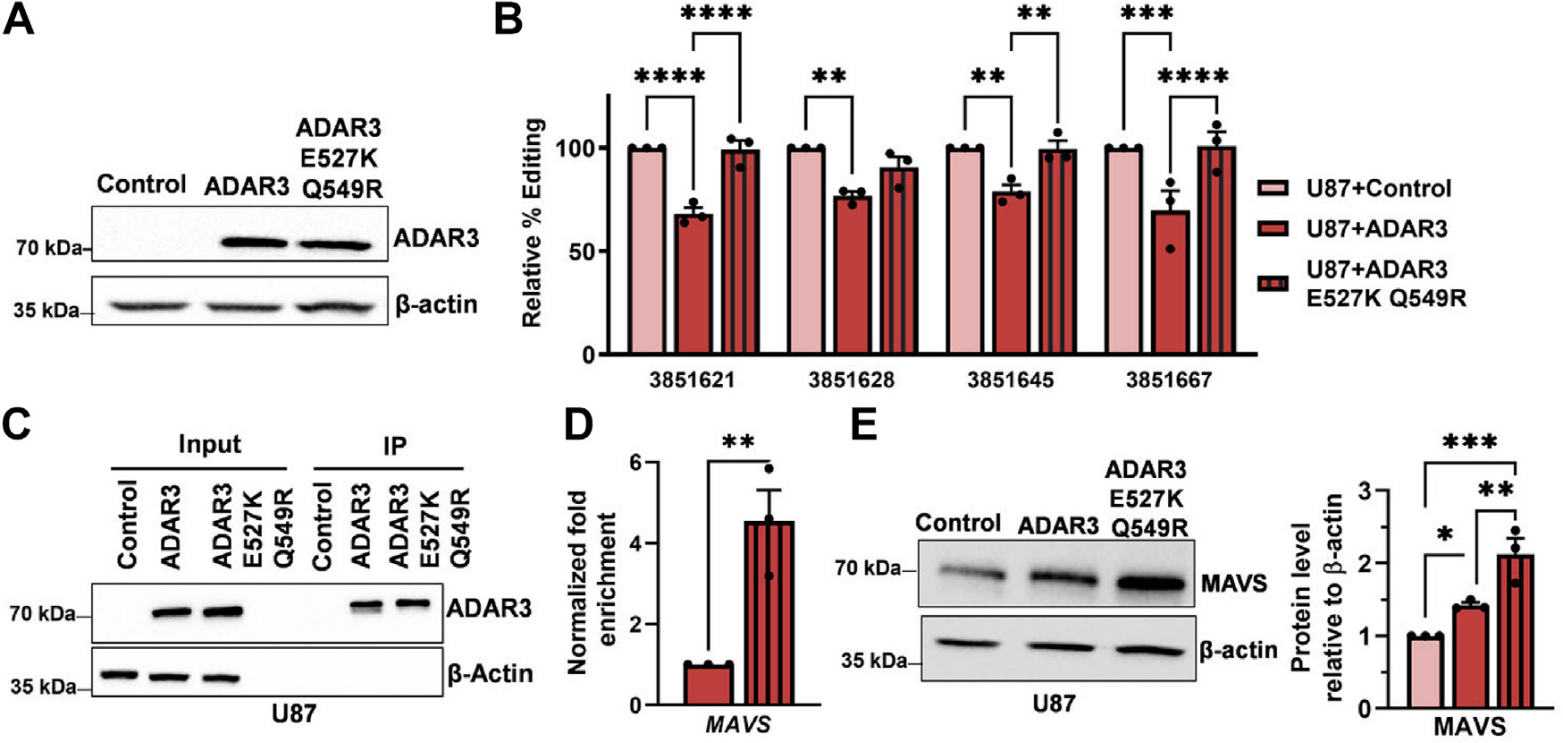
**ADAR3 regulates MAVS protein expression in a binding-dependent manner, independent of A-to-I editing**. *A*, equivalent amounts of lysate from the indicated cell lines were subjected to immunoblotting for ADAR3 and β-actin (n = 3 biological replicates). *B*, the relative editing level of the *MAVS* 3′ UTR in U87 cells expressing wildtype ADAR3 or ADAR3 E527K Q549R mutant compared with control cells. Two-way ANOVA followed by Sidak’s multiple comparisons tests, ∗∗*p* < 0.005, ∗∗∗∗*p* < 0.0001. *C*, equivalent amounts of lysate from the indicated cell lines were subjected to immunoprecipitation using FLAG magnetic beads. Input lysates from each cell line before incubation with beads (5% of lysate used per immunoprecipitation [IP]) and the corresponding protein immunoprecipitated (20% of each IP) were subjected to immunoblotting using antibodies against FLAG, ADAR3, and β-actin (n = 3 independent biological replicates). *D*, the bar graph represents the fold enrichment of *MAVS* cDNA in the IPs divided by cDNA in the input lysates normalized to the same ratio in U87 + ADAR3 cells. The mean of replicates is plotted with error bars representing SEM. Statistical significance was determined by two-tailed unpaired *t* test, ∗∗*p* <0.005. *E*, equivalent amounts of lysates from the indicated cell lines were immunoblotted using MAVS and β-actin antibodies. Bar graph represents the quantification of MAVS protein relative to β-actin. Statistical significance was determined by ordinary one-way ANOVA, ∗*p* ≤ 0.05, ∗∗*p* ≤ 0.005, ∗∗∗*p* ≤ 0.0005.

Stable expression of the ADAR3 E527K + Q549R mutant in U87 cells was performed with retroviral transduction alongside wildtype ADAR3 and the control cell line. Similar expression of wildtype ADAR3 and the ADAR3 E527K + Q549R mutant was confirmed by immunoblot analysis (Figs. 5*A* and S3*B*). The impact of the mutations in the ADAR3 deaminase domain on editing of the *MAVS* 3′ UTR was examined. Consistent with our initial results, expression of wildtype ADAR3 resulted in 20 to 25% reduced editing at multiple sites in the *MAVS* 3′ UTR (Fig. 5*B*). In contrast, expression of the ADAR3 E527K + Q549R mutant did not lead to a reduction in editing of these same sites in the *MAVS* 3′ UTR (Fig. 5*B*).

The lack of editing repression by the ADAR3 E527K + Q549R mutant could be due to an inability of this mutant to bind the *MAVS* transcript, a loss of editing repression function and/or a gain of deaminase function. To directly examine whether these mutations altered the ability of ADAR3 to bind RNA *in vivo*, an ADAR3 RIP-qPCR assay was performed. Equivalent amounts of wildtype ADAR3 and the E527K + Q549R mutant were immunoprecipitated (Fig. 5*C*). Compared with wildtype ADAR3, a nearly 5-fold increase in the enrichment of *MAVS* mRNA was observed for the ADAR3 E527K + Q549R IPs (Fig. 5*D*). Together, these data indicate that the lack of editing repression observed in the ADAR3 E527K + Q549R mutant is not a result of a lack of ADAR3 E527K + Q549R interaction with the *MAVS* transcript.

As the ADAR3 E527K + Q549R protein exhibited strong binding to the *MAVS* transcript and the expression of this mutant led to similar editing levels in the *MAVS* 3′ UTR compared with U87 control cells, the effect of ADAR3 E527K + Q549R on MAVS protein expression was examined. As a positive control, the impact of wildtype ADAR3 on MAVS protein expression was also examined. Consistent with previous results, ADAR3-expressing cells had significantly higher MAVS protein expression compared with control U87 cells (Fig. 5*E*). Compared with the wildtype ADAR3-expressing cells, the U87 cells expressing the ADAR3 E527K + Q549R mutant exhibited significantly higher MAVS protein expression (Fig. 5*E*) but did not alter *MAVS* mRNA expression (Fig. S3*C*). Consistent with our previous results, the level of editing showed no correlation with MAVS protein expression. Together these results indicate that ADAR3 regulates MAVS protein expression in a binding-dependent manner.

## Discussion

Altered ADAR expression and aberrant editing have been identified in many cancers, with most focus on the functional deaminases ADAR1 and ADAR2 (62, 63). Prior studies indicate that the editing activity of ADAR1 and ADAR2 can be regulated by ADAR3, the deaminase-deficient ADAR family member, *in vitro* and for at least one transcript in a brain cancer cell line (12, 25). However, the cellular targets of ADAR3 remain largely unexplored. In this study, we identified the RNA targets of ADAR3 in the U87 glioblastoma cell line and examined the global effects of ADAR3 expression on RNA editing. Our results indicate that ADAR3 regulates RNA editing across the transcriptome and inhibition of editing within transcripts correlates with ADAR3 binding. In addition, we demonstrate that ADAR3 has the potential to alter gene expression at the protein level in a binding-dependent manner, independent of its role as a negative regulator of A-to-I editing.

Our transcriptome-wide analysis revealed that ADAR3 expression resulted in a global reduction in A-to-I editing and that most differentially edited sites are in ADAR3-bound transcripts. Together, these data indicate that ADAR3 acts as an important negative regulator of editing. However, it is important to note that less than 10% of ADAR3-bound transcripts exhibit differential editing, suggesting that RNA binding by ADAR3 could have gene regulatory functions independent of altering editing. It is possible that ADAR3 could influence gene expression of a number of these targets in a binding-dependent manner, as reported for some targets of ADAR1 and ADAR2 (36, 64, 65). Consistent with this, we observed that ADAR3 can regulate MAVS protein expression without affecting *MAVS* mRNA levels. Future work focused on both mRNA and protein expression analysis is required to determine whether ADAR3 binding to transcripts is a widespread mechanism to regulate gene expression. However, since ADAR3-mediated editing repression correlates with RNA binding, mutation of the ADAR3 RNA binding domains may alter editing levels in some target RNAs. Therefore, future studies aimed at distinguishing between these gene regulatory functions of ADAR3 should also determine if the presence or absence of ADAR1 and ADAR2 impacts the gene regulatory function of ADAR3.

In addition to the editing changes mediated by ADAR3 in shared targets with ADAR1 and ADAR2, in this study, we identified over 1300 unique ADAR3-bound transcripts. As ADAR3, but not ADAR1 or ADAR2, possesses an arginine-rich (R) domain that provides single-stranded RNA binding activity *in vitro* (12), it is possible that this domain could provide ADAR3 a unique means to recognize specific targets *in vivo*. In addition to RNA binding, both the R-domain and dsRNA binding domain have been shown to facilitate protein–protein interactions in other RNA-binding proteins (66, 67). Therefore, it is possible that these domains of ADAR3 facilitate its association with other proteins, which in turn recruit ADAR3 to specific targets. Along with RNA binding domains, the deaminase domain could also contribute to RNA binding/recognition. In fact, within the deaminase domain, a highly conserved 24-amino-acid sequence referred to as the RNA-binding loop has been shown to regulate both ADAR1 and ADAR2 editing activity and substrate selectivity for specific adenosines (61, 68). However, to date, mutations in the RNA-binding loop and/or other regions of the deaminase domain have not been shown to alter RNA binding affinity of ADARs. Interestingly, our analysis of the ADAR3 protein with two mutations in the deaminase domain, E527K and Q549R, demonstrated a significantly enhanced ability to bind RNA in cells when compared with wildtype ADAR3. It would be interesting in future studies to see if this mutant also imparts differences in binding to specific target RNAs and to determine how the individual domains of ADAR3 influence target recognition, editing repression, and gene expression.

To understand how ADAR3 binding could alter gene expression, we focused on dissecting the mechanism of how ADAR3 regulates one specific transcript, which encodes the immune response regulator, MAVS. We observed that ADAR3 binds and inhibits ADAR1-mediated editing in the *MAVS* 3′ UTR. However, unlike ADAR1, our data indicate that ADAR3 binding to the *MAVS* transcript upregulates MAVS protein expression in an editing-independent manner. This suggests that, even in shared targets, ADARs could have unique regulatory roles. Interestingly, ADAR3 expression had no effect on the *MAVS* mRNA level, indicating that ADAR3-mediated regulation occurs at the posttranscriptional or translational level. A recent study showed that the interferon-inducible ADAR1 p150 isoform edits the *MAVS* 3′ UTR and reduces *MAVS* mRNA stability by influencing recruitment of the stabilizing RBP, HuR, in HepG2.2.15 cells (35). Another study in HeLa cells reported destabilization of *MAVS* mRNA through HuR binding to AU-rich elements in the *MAVS* 3′ UTR (37). In addition to A-to-I editing, N^6^-methyladenosine (m^6^A) modification of the *MAVS* 3′ UTR regulates nuclear retention and translation efficiency to MAVS (38). Thus, it is possible that, in addition to repressing ADAR1 editing activity, ADAR3 binding to the *MAVS* 3′ UTR may block or recruit other RBPs that could regulate MAVS protein expression. Alternatively, the *MAVS* 3′ UTR harbors several miRNA-binding sites (57) and the presence of ADAR3 could inhibit miRNA binding thus altering translation efficiency. Another possibility is that ADAR3 could alter the expression or activity of a regulatory protein, which in turn regulates MAVS, and thus, while ADAR3 binding is important, the influence of ADAR3 on MAVS protein expression is indirect. Future studies are necessary to dissect the mechanism of editing-independent gene regulation by ADAR3.

Our findings on ADAR3-mediated MAVS regulation may provide an insight into a novel regulatory mechanism of brain-specific ADAR3 in neuronal homeostasis and disease. Recent studies show that MAVS can promote a neuroinflammatory tumor microenvironment and is a potential therapeutic target in glioma treatment (69). Although MAVS is widely studied for mediating the activation of NF-κB and IRF3 in response to viral infection (29), MAVS also plays a critical role in central nervous system inflammation and metabolism. The interaction of MAVS with cytosolic phospholipase A2 (cPLA2) promotes a proinflammatory transcriptional program associated with pathogenesis in neurologic diseases such as experimental autoimmune encephalomyelitis and multiple sclerosis (70). In addition, MAVS controls glycolysis by regulating hexokinase 2 activity, which can have neurotoxic effects through reduced lactate release (71). Moreover, MAVS is reported to be a key signaling molecule and potential target for treatment in microglia-driven inflammatory brain diseases (72). As aberrant ADAR3 expression has been connected to several neurological diseases, the ability of ADAR3 to alter MAVS expression and signaling should be explored. Furthermore, in addition to *MAVS*, we found 80 ADAR3-bound genes that overlap with the Kyoto Encyclopedia of Genes and Genomes (KEGG) pathway of neurodegeneration (hsa05022, *p* value = 0.03). More specifically, the KEGG analysis also revealed a significant enrichment of genes involved in AD (hsa05010, *p* value = 0.0045) and identified 71 ADAR3-bound transcripts in this category. As both altered sequence and aberrant *ADAR3* expression have been reported in patients with AD (20, 21, 22), future studies should focus on whether these 71 ADAR3-bound transcripts have altered expression upon changes in ADAR3 levels. Furthermore, two ADAR3-bound transcripts we identified in this study, *ORAI2* and *STY11*, were recently identified from brain tissue samples as transcripts where the editing level in the 3′ UTR correlated with the degree of dementia in individuals with AD (22). Unfortunately, we are unable to discern the impact of ADAR3 on editing of these transcripts from our analysis of RNA isolated from U87 glioblastoma cell lines, as little editing of *STY11* is observed in these cells and read counts for *ORAI2* are very low. Future studies to determine whether the ADAR3 interactions with these transcripts influence gene expression in a binding- or editing-dependent manner could improve our understanding of contribution of ADAR3 in neuronal disorders.

In addition to binding-dependent regulation of gene expression, our study suggests that an ADAR3 protein containing mutations in two key residues in the deaminase domain, E527 and Q549, may allow ADAR3 to exhibit a gain of deaminase function in cells. These amino acids were previously identified among the five residues (E527Q, A389V, V485I, Q733D, and Q549R) important for generating an ADAR3 protein that exhibited deaminase activity *in vitro* and in human cell lines (68). To directly examine the deaminase activity of the ADAR3 E527K + Q549R mutant, editing assays should be performed in cells lacking both ADAR1 and ADAR2 or *in vitro* using recombinant ADAR3 E527K + Q549R protein. Our decision to generate the ADAR3 E527K mutant instead of the E527Q mutation used previously was due to ADAR3 E527K being a highly recurrent, patient-derived tumor missense mutation. In the COSMIC database, there are 3052 somatic mutations within the coding region of ADAR3. In contrast, human ADAR1 and ADAR2 only have 730 and 1033 coding mutations, respectively. When focusing on recurrent mutations (occurring in three or more patient samples), this trend is still apparent, with twice as many missense mutations in the ADAR3 coding region compared with ADAR1 and ADAR2 (27/332, 16/214 and 13/322, respectively). Interestingly, ∼74% of recurrent ADAR3 missense mutations are mapped to the inactive deaminase domain, whereas only ∼46% of ADAR1 and ∼68% of ADAR2 recurrent mutations map to the deaminase domain (Fig. S4). Several mutations were highly recurrent (≥3 patients) in the ADAR3 deaminase domain and were analogous to functionally important ADAR2 residues. It will be interesting to examine whether one or more of these recurrent mutations together could alter ADAR3 deaminase activity and/or *in vivo* RNA binding. We also noticed that all patient samples with the ADAR3 E527K mutation involved skin as the primary tissue of origin, with most coming from malignant melanoma and one from a squamous cell carcinoma tumor. As ADAR3 expression is restricted to nervous tissue (12), most cancer studies focused on ADAR3 have been limited to glioblastoma (25, 26, 27, 28). However, based on the COSMIC mutations and our results, future studies should examine the consequence of altered ADAR3 expression, RNA binding, and potential gain of deaminase activity in other cancers.

## Experimental procedures

### Cell culture and transfection

U87 cells were grown in Dulbecco’s modified Eagle’s medium (Mediatech) supplemented with 10% fetal bovine serum (FBS) (Sigma), 100 μg/ml penicillin, and 100 units/ml streptomycin (Mediatech). U373 and GBM43 cells (a kind gift from Karen Pollok, IUSM) were grown in Iscove's modified Dulbecco's medium (IMDM) (Gibco) supplemented with 10% FBS, 100 μg/ml penicillin, and 100 units/ml streptomycin (Mediatech) and Dulbecco's modified Eagle's medium with 10% FBS, 100 μg/ml penicillin, 100 units/ml streptomycin, and 10 mM Hepes buffer (Gibco). ADAR1 knockdown and scrambled shRNA U87 cells were generated by lentiviruses produced in the laboratory as described (73). Transduced U87 cells were selected with 0.075 mg/ml puromycin, and ADAR1 knockdown was confirmed by immunoblot using an ADAR1 antibody (a kind gift from Brenda Bass, University of Utah). Infection by retroviruses produced in the laboratory was used to express 3xFLAG:ADAR3, 3XFLAG:ADAR1, and 3XFLAG:ADAR2 as described (25). After selection, cells were maintained in 0.2 mg/ml G418. Expression of 3xFLAG:ADAR3 was confirmed by Western blot using anti-FLAG antibody (Sigma, F-1804). The Universal Mycoplasma Detection Kit (American Type Culture Collection) was used to verify that all cell lines are free from mycoplasma contamination.

### RNA isolation and qPCR

Total RNA was isolated using TRIzol (Invitrogen) and further purified by treatment with TURBO DNase (Ambion) followed by the RNeasy Extraction kit (Qiagen). For qPCR, synthesis of cDNA was performed using Superscript III (Invitrogen) with random hexamer (Fisher Scientific) and oligo-dT (Fisher Scientific) or gene-specific reverse transcription primers (Table S4). After reverse transcription, 20 μl of water was added to the cDNA. Gene expression or enrichment for binding was determined using SybrFast Master Mix (KAPA) and gene-specific primers (Table S4) on a ThermoFisher Quantstudio 3 instrument.

### Library preparation and sequencing of polyadenylated RNA

Libraries were created from RNA isolated from three independent biological replicates of U87 cells expressing an empty vector and U87 cells expressing 3XFLAG:ADAR3. PolyA+ beads (Invitrogen) were used to select for mRNA, and libraries were generated using the KAPA Strand-Specific RNA Library Kit according to the manufacturer’s instructions. Sequencing was performed by the Indiana University School of Medicine Center for Medical Genomics on an Illumina NextSeq500 instrument. In brief, 70 to 100 million, 75-bp paired-end, RNA-Seq reads were trimmed of adapters and aligned to hg19 (gencode release 28) using the following STAR (v2.5.2b) parameters: *[*--outFilterMismatchNmax 999 --outFilterMismatchNoverLmax .3 --outFilterMismatchNoverReadLmax .2 --outFilterMultimapNmax 1*]*. Mapped reads limited mismatches to <20% of the read length and mapping to only one location.

### SAILOR

*De novo* editing site identification was performed using *SAILOR* (45) for three biological replicates of each cell line. Known SNPs were removed at this step using the list of common SNPs from dbSNP (Build 151) and a list of known SNPs in the U87 cell line (74). Sites were considered an editing site based on three criteria: (1) the *SAILOR* prediction was ≥0.99 confidence, (2) the coverage of the site was ≥20 reads, and (3) the site was identified in all three biological replicates of the cell line analyzed.

### Variant analysis

Variant sequences in the reads present at editing sites identified by *SAILOR* were quantified using Samtools (v1.3.1) mpileup against the Gencode hg19 reference genome. Initially, duplicate reads were removed from each sample using samtools rmdup. Mpileup was then run on the remaining reads in each sample using the parameters [-u -t DP4 -v -l]. The DP4 field was used to determine read coverage and editing percentage at each site. The effects of ADAR3 on the variant population (percent editing) were calculated by pairwise comparison of the editing levels in U87 cells expressing ADAR3 to the control cells for each biological replicate.

### ADAR3 RNA immunoprecipitation assays

3XFLAG:ADAR3 RIP was performed in U87 cells, and the endogenous ADAR3 RIP assay was performed in U373 cells as described (75). Briefly, for both assays, cells were washed with 1X PBS (137 mM NaCl, 2.7 mM KCl, 10 mM Na_2_HPO_4_, 2 mM KH_2_PO_4_) and subjected to 150 mj/cm^2^ of UV radiation using the Spectrolinker (Spectronic Corp). Cells were then resuspended in a wash buffer (20 mM Tris-HCl, pH 7.5, 500 mM NaCl, 10 mM EDTA + 10% glycerol, 0.1% NP-40, 0.5% Triton X-100) and sonicated on ice. The crude lysate was centrifuged to remove insoluble material, and the cleared lysates were added to either FLAG magnetic beads (Sigma) or Dynabeads M-280 Sheep anti-Rabbit IgG beads (Invitrogen) preincubated with affinity-purified ADAR3 antibody. After 1 h incubation at 4 °C, the magnetic beads were washed with ice-cold wash buffer and resuspended in 1X TBS (16 mM Tris-HCl, pH 7.5, 110 mM NaCl). RNasin (Promega) and proteinase K (Sigma) were added and incubated at 42 °C for 15 min to degrade protein and release ADAR3-bound RNA. The protein samples were subjected to immunoblotting and the RNA samples were isolated (as described above) and subjected to either high-throughput sequencing or qPCR (as described above).

### Library preparation and sequencing of RIP samples

For high-throughput sequencing, RNA-Seq libraries were prepared using the Clontech pico v2 kit stranded-RNA library preparation kit according to the manufacturer’s instructions (Indiana University Center for Medical Genomics). Libraries were constructed using a ribo-erase step to remove ribosomal RNA and random hexamer primed reverse transcription to identify ADAR3-bound RNAs. The samples were sequenced on an Illumina NextSeq500 as 2 × 75 bp paired end reads. In total, 12 RNA-Seq datasets were generated, and each had approximately 24 to 36 million reads, except for one of the control IPs, which had 163 million reads.

### Identifying ADAR3-bound targets

In brief, 2 × 75 bp paired-end, RNA-Seq reads were trimmed of adapters and aligned to hg19 (gencode release 28) using the following STAR (v2.5.2b) parameters: *[*--outFilterMismatchNmax 999 --outFilterMismatchNoverLmax .3 --outFilterMismatchNoverReadLmax .2 --outFilterMultimapNmax 1*]*. These parameters allowed for mapping of reads where the total mismatches did not exceed 20% of the read length and alignment to only one genomic location. FeatureCounts (v1.5.2) was used to count mapped reads to Gencode hg19 release 28 gene annotation. Genes with zero read counts across all samples were removed as indicating nonexpressed transcripts. To determine enrichment of transcripts in the IP, RNA-Seq datasets for three biological replicates of both IP and input samples for each cell line were analyzed. Raw read counts were input into DESeq2 (v1.18.1) to test for ratio of ratios, in this case ((IPA/Input A)/(IPB/Input B)), using a likelihood ratio test. Transcripts with a log_2_ fold change ≥0.5 and a p-adjusted value <0.05 between ADAR3-expressing cells and controls were considered ADAR3 bound (Table S1*B*). A secondary computational analysis of ADAR3-bound targets was performed at the IUSM Center for Computational Biology and Bioinformatics. This analysis used the same alignment parameters and mapping as described above. Raw read counts were input into EdgeR and fit using the generalized linear model to determine the enrichment of transcripts in the IPs. Transcripts with a false discovery rate ≤0.05 between ADAR3-expressing cells and controls were considered ADAR3 bound (Table S1*C*).

### Western analysis in U87 cells

U87 cells were plated at a density of 2 × 10^5^ cells/ml/plate. After 24 h, the medium was removed and washed with 1X PBS. The cells were trypsinized and centrifuged at 1200*g* for 5 min. The cell pellet was washed with cold 1× PBS and resuspended in lysis buffer (2% SDS, 50 mM Tris-HCl, 10% Glycerol) with protease inhibitor (Roche) and kept on ice. The cells were sonicated and centrifuged at 15,000 rpm for 10 min. The protein concentration of the supernatant was determined by the Bradford assay. DTT (0.1 M) and Bromophenol blue (0.1%) were added, and lysates were boiled for 5 min. An equivalent amount of protein lysates was subjected to SDS-PAGE and immunoblotting using antibodies against MAVS (Cell Signaling, 39935), ADAR1 (a kind gift from Brenda Bass), and β-actin (Cell Signaling, 8457S). ADAR3 antibody was generated (Cocalico Biologicals) against the N-terminal region of human ADAR3 (amino acids 2–102) fused to glutathione-*S*-transferase (GST). The first 303 nucleotides (101 amino acids) in the N-terminal region of ADAR3 were PCR amplified from a plasmid containing the ADAR3 coding sequence using primers listed in Table S3. The PCR product was then cloned into a pGEX-KG vector with BamHI/XbaI to generate a GST fusion protein. Recombinant protein expression was induced using IPTG in *Escherichia coli pRIL*, and the soluble protein was then purified using glutathione agarose resin. The purified GST-ADAR3 N-terminal protein was used to immunize rabbits (Cocalico Biologicals), but preimmune serum was obtained before antigen administration. The polyclonal antiserum was first purified by removing GST antibodies using the blot affinity purification method as described (76) and the recombinant GST protein was purified as described for the immunogen. The precleared antiserum was then used for blot affinity purification of antibodies using the ADAR3 N-terminal GST fusion protein. Specificity of the affinity purified antibody for endogenous ADAR3 was tested by comparing lysates from glioblastoma cell line GBM43, which expresses endogenous ADAR3 and U87 cells expressing 1X/3XFLAG:ADAR3 (Fig. S5*A*). The ADAR3 antibody was tested for cross-reactivity with ADAR1 and ADAR2 by immunoblotting lysates from 3XFLAG:ADAR1 and 3XFLAG:ADAR2-expressing U87 cells (Fig. S5*B*).

### Reverse transcription and Sanger sequencing editing assays

RNA was extracted as described above. Reverse transcription was carried out using Superscript III reverse transcriptase (Invitrogen). Briefly, 2 μg of DNase-treated RNA, gene-specific primers (250 nM) (Table S4), and dNTPs (0.5 mM each) were incubated at 65 °C for 5 min followed by 1 min on ice. A reaction mixture of 200 units of Superscript III, first-strand buffer (1×), 40 units of RNAsin (Promega), and 5 mM DTT was added. The reaction was incubated at 55 °C for 1 h followed by heat inactivation for 15 min at 70 °C. The reaction mixture was incubated with 5 units of RNase H (NEB) at 37 °C for 20 min. The resulting cDNA and genomic DNA were amplified using PFX Platinum DNA Polymerase (Invitrogen) or Phusion high fidelity DNA Polymerase (NEB) using primers spanning the region of interest. The PCR products were resolved by agarose gel electrophoresis, excised from the gel, and purified using a Qiagen Gel purification kit. The gel-purified DNA was subjected to Sanger sequencing, and the chromatograms were aligned using CodonCode Aligner. The height of A and G peaks at each editing site using Adobe photoshop and level of editing was calculated using the formula: % editing = height of G peak/(height of A peak + height of G peak) ∗100. Negative controls without Superscript III were conducted for all editing assays to ensure that DNA sequenced resulted from cDNA amplification.

### Site-directed mutagenesis

ADAR3 deaminase domain mutants were generated by a two-step PCR site-directed mutagenesis with the primers (IDT) listed in Table S4. Primers carrying the Q549R mutations and restriction enzyme sites were used to amplify the ADAR3 from plasmid pLNCX2 3XFLAG:ADAR3. The PCR product was digested using BamHI and XhoI and ligated into the pLNCX2 vector. The ADAR3 Q549R mutant was then used as the template for PCR amplification using E527K mutagenic primers and ligated into pLNCX2. All plasmids generated were confirmed by Sanger sequencing.

## Data availability

High-throughput sequencing data can be accessed at the Gene Expression Omnibus (GSE154864).

## Supporting information

This article contains supporting information (49, 50, 51).

## Conflict of interest

The authors declare that they have no conflicts of interest with the contents of this article.
