# Supplementary material for: RNA binding by ADAR3 inhibits adenosine-to-inosine editing and promotes expression of immune response protein MAVS
Source: J Biol Chem. 2022 Jul 15;298(9):102267. doi: 10.1016/j.jbc.2022.102267 (PMC9418441; doi:10.1016/j.jbc.2022.102267)
Supplement: Supporting information [file mmc1.pdf]

Supporting information

RNA binding by ADAR3 inhibits adenosine-to-inosine editing and promotes expression of immune response protein MAVS

Reshma Raghava Kurup, Eimile K. Oakes, Aidan C. Manning, Priyanka Mukherjee, Pranathi Vadlamani and Heather A. Hundley

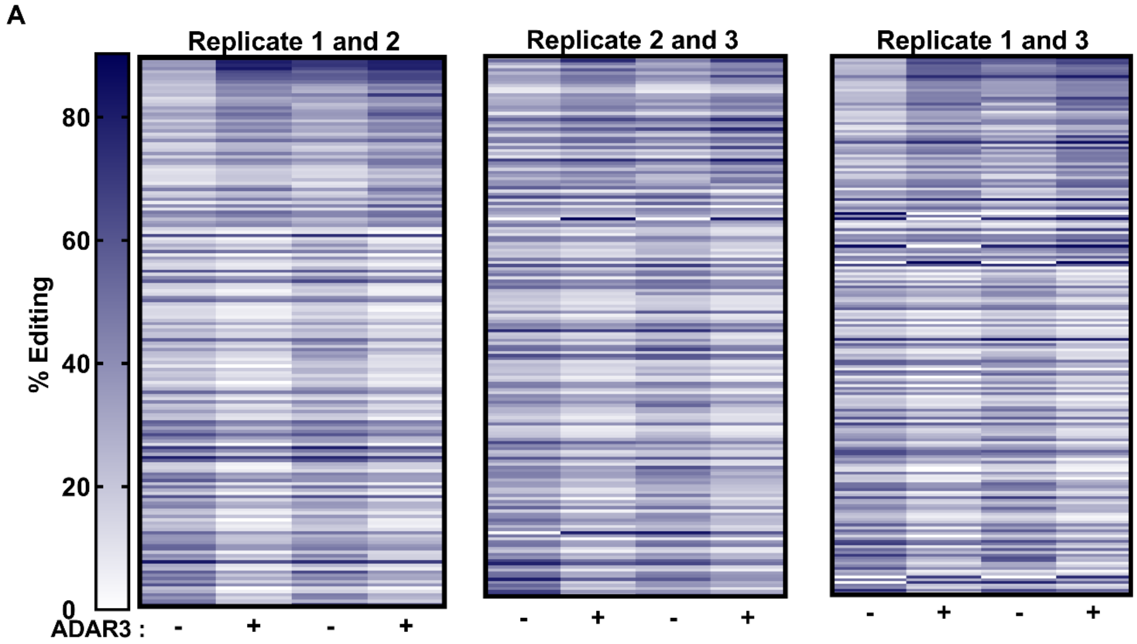

Figure S1: A, Heat map of the percent editing (colored in increasing shades of navy blue) determined from variant calling of two independent biological replicates of RNA-seq data. The specific datasets for each image are mentioned above the individual heat maps. Each line represents an individual editing site identified from the RNA-seq data of the U87 cell lines indicated on the x-axis.

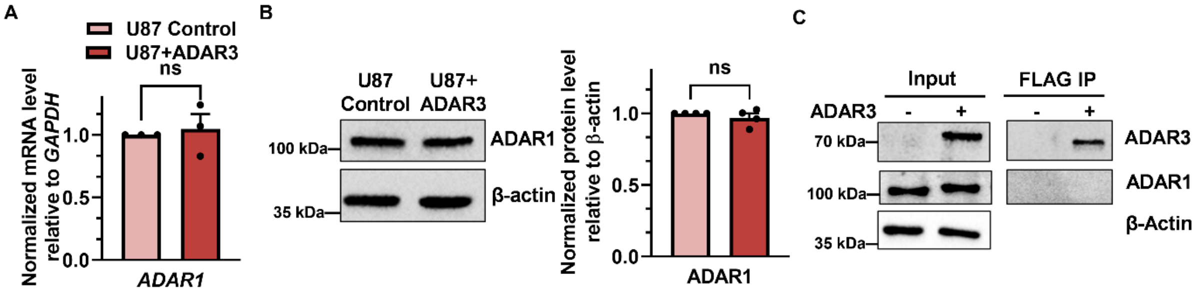

Figure S2: A, qPCR quantification of the *ADAR1* mRNA level normalized to *GAPDH* in control and ADAR3-expressing U87 cells. The mean of three biological replicates is plotted with SEM. Statistical significance was determined using a two-tailed unpaired t-test, ns = $p > 0.05$. B, Equivalent amounts of lysates from the indicated cell lines were subjected to immunoblotting for ADAR1 and β-actin (n= 4 biological replicates). Bar graph represents the quantification of ADAR1 (p110) protein relative to β-actin. Statistical significance was determined using a two-tailed unpaired t-test, ns = $p > 0.05$. C*,* Lysate (input) from control and ADAR3-expressing U87 cells (5% of lysate used per IP) and the corresponding samples immunoprecipitated using FLAG magnetic beads (10% of IP) were subjected to immunoblotting using antibodies against, ADAR1, ADAR3, and β-actin. Blot is representative of three independent biological replicates*.*

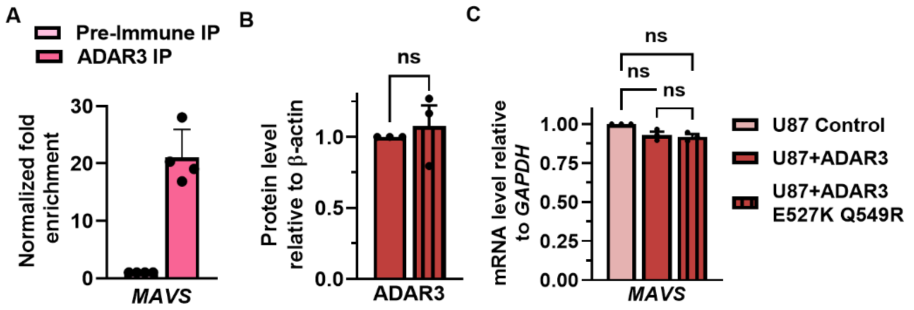

Figure S3: A, Bar graph represents the fold enrichment of cDNA in the ADAR3 IPs normalized to pre-immune IP in U373 cells. The mean of four biological replicates is plotted with error bars representing SEM. Statistical significance determined by two tailed unpaired t-test, ***p < 0.0001. B, Bar graph represents the quantification of ADAR3 protein expression relative to β-actin in U87 cells expressing the ADAR3 E527K Q549R mutant normalized to wildtype ADAR3-expressing U87 cells (n=3 biological replicates). A two tailed unpaired t-test was performed to determine statistical significance, ns representing p > 0.05. C, qPCR quantification of the *MAVS* mRNA level normalized to *GAPDH* in control and ADAR3-expressing U87 cells. The mean of three biological replicates is plotted with SEM. Statistical significance was determined by one-way ANOVA, ns representing p > 0.05.

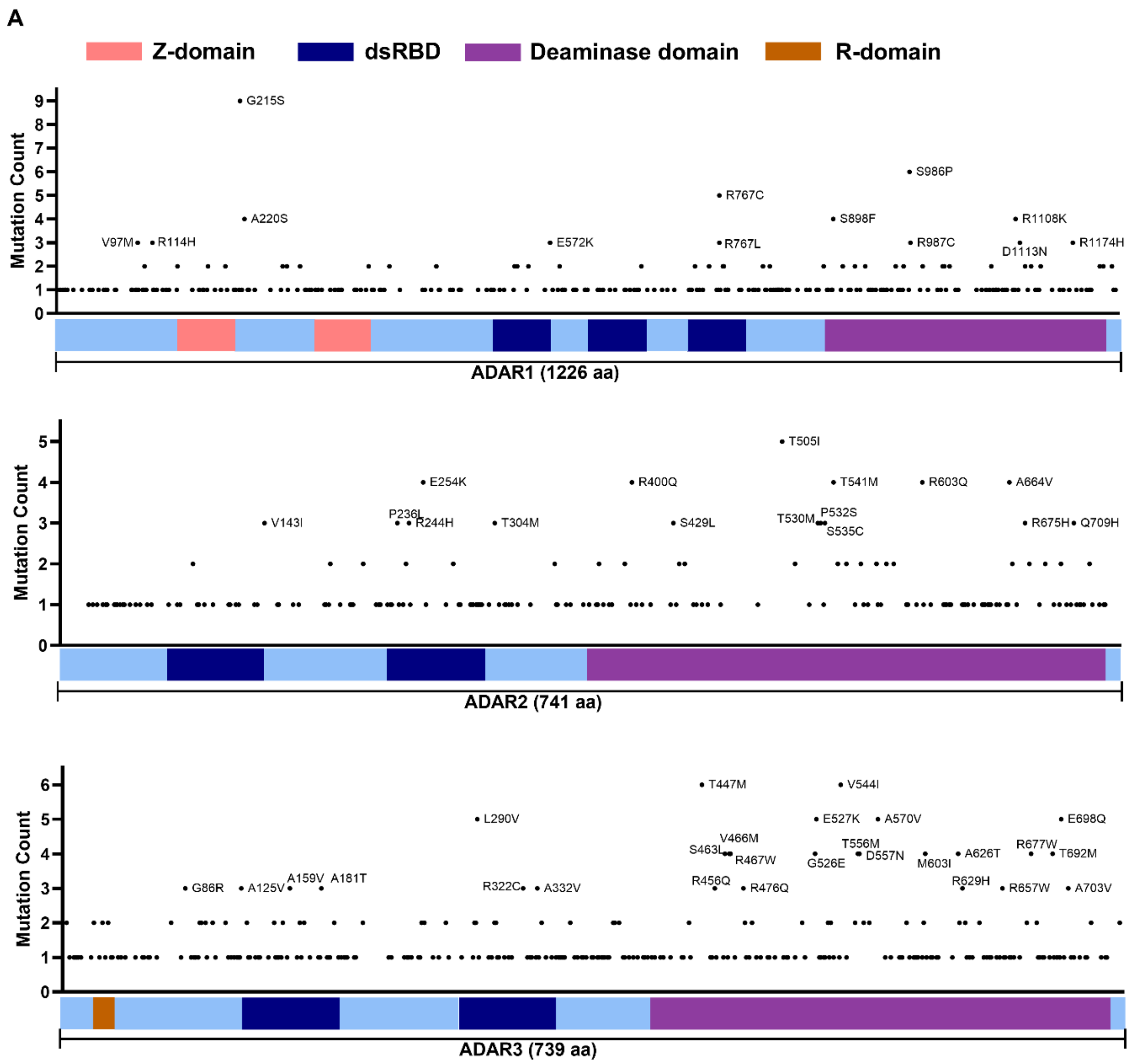

Figure S4: Graphical summary of missense mutations in ADAR1, ADAR2, and ADAR3 identified from the COSMIC database (v95). The position of the missense mutations on the protein domain is plotted against the number of patient samples carrying the mutation. The amino acid change for recurrent missense mutations (≥3 patient samples) is marked on the graph.

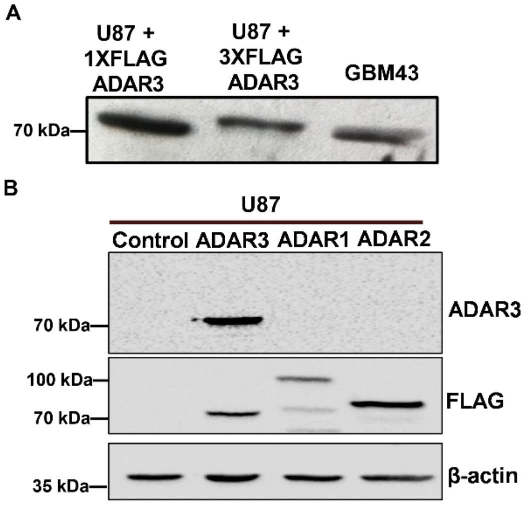

Figure S5: A, Lysates from U87 cells expressing 1X or 3X FLAG:ADAR3 and GBM43 glioblastoma cells were immunoblotted with our custom ADAR3 antibody. B, Equivalent amounts of lysate from U87 cells transduced with a retrovirus where no human gene is expressed from the CMV promoter (control) or 3X FLAG:ADAR3 or 3X FLAG:ADAR1 or 3X FLAG:ADAR2 were subjected to immunoblotting with FLAG, ADAR3 and β-actin antibodies.
